# Metabolomics reveals biotic and abiotic elicitor effects on the soft coral *Sarcophyton ehrenbergi* terpenoid content

**DOI:** 10.1038/s41598-017-00527-8

**Published:** 2017-04-05

**Authors:** Mohamed A. Farag, Dalia A. Al-Mahdy, Achim Meyer, Hildegard Westphal, Ludger A. Wessjohann

**Affiliations:** 1 Pharmacognosy department, College of Pharmacy, Cairo University, Cairo, Kasr El Aini st., P.B. 11562 Egypt; 2 Leibniz Centre for Tropical Marine Research, Fahrenheit Str.6, D-28359 Bremen, Germany; 3 Bremen University, Bremen, Germany; 4 Leibniz Institute of Plant Biochemistry, Dept. Bioorganic Chemistry, Weinberg 3, D-06120 Halle (Saale), Germany

## Abstract

The effects of six biotic and abiotic elicitors, i.e. MeJA (methyl jasmonate), SA (salicylic acid), ZnCl_2_, glutathione and *β*-glucan BG (fungal elicitor), and wounding, on the secondary metabolite accumulation in the soft coral *Sarcophyton ehrenbergi* were assessed. Upon elicitation, metabolites were extracted and analysed by ultra-performance liquid chromatography-mass spectrometry (UPLC-MS). Except for MeJA, no differences in photosynthetic efficiency were observed after treatments, suggesting the absence of a remarkable stress on primary production. Chemometric analyses of UPLC-MS data showed clear segregation of SA and ZnCl_2_ elicited samples at 24 and 48 h post elicitation. Levels of acetylated diterpene and sterol *viz*., sarcophytonolide I and cholesteryl acetate, was increased in ZnCl_2_ and SA groups, respectively, suggesting an activation of specific acetyl transferases. Post elicitation, sarcophytonolide I level increased 132 and 17-folds at 48 h in 0.1 mM SA and 1 mM ZnCl_2_ groups, respectively. Interestingly, decrease in sarcophine, a major diterpene was observed only in response to ZnCl_2_, whereas no change was observed in sesquiterpene content following treatments. To the best of our knowledge, this study provides the first documentation for elicitation effects on a soft corals secondary metabolome and suggests that SA could be applied to increase diterpenoid levels in corals.

## Introduction

Marine invertebrates are well recognized for their novel secondary metabolites composition endowed with a wide range of structural diversity and various biological activities. Among them, soft corals constitute an important class of marine invertebrates which have sophisticated biochemical as well as physiological mechanisms, enabling them to produce elaborate bioactive compounds for purposes such as survival of various stresses, protection against predation, competition, or chemical communication in symbiotic relationships^1^.

Soft corals belonging to genus *Sarcophyton* (subclass Octocorallia; order Alcyonaceae; family Alcyoniidae)^2, 3^ are among the largest biodiversity contributors to many tropical coral habitats including the Red Sea and the Indo Pacific region^4, 5^. A wealth of unique secondary metabolites including diterpenes, sesquiterpenes and sterols have been isolated and identified in different soft coral species^6, 7^. Diterpenes, mainly cembranoids are the most abundant metabolites identified in genus *Sarcophyton* and they are considered the main chemical defence of corals against natural predators^8–10^. Cembranoids contain a 14-membered macrocyclic skeleton which usually exhibit cyclic ether, lactone or furan moieties around the cembrane ring^11^. From a biomedical perspective, cembranoids possess a myriad of biological effects including cytotoxicity^12, 13^, anti-inflammatory^14^, neuro-protective^15^, antimicrobial^16^ and antiviral activities^17^.

Despite the fact that several diterpenes from *Sarcophyton* species are promising drugs with potential for biomedical applications, such as sarcophine^17^, sarcophytol^18^ and sarcophytolide^19^, the inadequate supply of coral biomass as a raw material has delayed the development of these agents. For drug development or production, reliance on soft coral reef is not enough to secure industrial demand and it would be as well environmentally destructive to supply drugs by harvesting of soft corals^20, 21^. As with terrestrial plants, *ex-situ* culture of soft corals affords an alternative for the natural production of soft coral bioactive metabolites. *Ex-situ* or *in vitro* cultures parameters could be optimized to enhance product levels including culture temperature, light intensity, nutrients composition and possibly enabling the manipulation with different biotic and abiotic factors that could maximize coral secondary metabolite production^22^.

Elicitation *i.e*. treatment of *in vitro* cultures with biotic and abiotic elicitors, is considered an important tool towards enhanced production of desirable products^23^. Considering that most secondary metabolites are produced naturally in response to pathogen defence or stress response, elicitors are external stimuli intended to mimic these responses, transiently inducing the accumulation of target metabolites^24^. Biotic elicitors are derived from biological origin such as (signalling or surface) compounds from fungi, bacteria, plant cell wall fragments. In contrast, abiotic elicitors include chemical and physical stressors *i.e*., salts of heavy metals, inorganic and synthetic compounds or even UV-radiation^25, 26^. One of the most relevant stressors inducing elicitation is wounding, whereby e.g. plants can distinguish scratches from the bites of enemies^27^. Both biotic and abiotic elicitors have the potential to enhance the production of terrestrial plant defence related metabolites from almost all chemical classes, such as sesquiterpene lactones^28^, anthocyanins^29^ and flavonoids^30^. Methyl jasmonate (MeJA) and salicylic acid (SA) are two key signalling molecules known to elicit defence related responses in planta during herbivore predation mostly *via* the production of defence secondary metabolites^31^. *β*-Glucan is an oligosaccharide derived from cell walls of fungi, while glutathione is a tripeptide required for efficient defence against pathogens and redox/electrophile disbalances^23^.

Several studies were reported regarding elicitation of terpenes in terrestrial plants, with most reports indicating that these natural products are rather inducible. Several groups have demonstrated the increased production of the diterpenoid Paclitaxel (Taxol™) and other taxanes after elicitation with MeJA in cell cultures of *Taxus chinensis* and *T. baccata* ^32, 33^. MeJA was found to mimic fungal elicitation and mechanical wounding in the Norway Spruce *Picea abies* L. Karst *via* upregulating terpenoid resin biosynthesis^34^ whereas the polysaccharide fraction of yeast extract, Ag^+^ and Cd^2+^ were identified as inducers of tanshinone diterpene production in *Salvia miltiorrhiza* ^35^. Meanwhile, very few reports could be traced regarding elicitation of terpenes in soft corals; one study stated that decreased levels of UV/visible radiation induced the production of pseudopterosin diterpene glycosides in the gorgonian sea whip *Pseudopterogorgia elisabethae* ^36^ and another study reported that MeJA induced production of fuscol within a dinoflagellate preparation of the coral *Eunicea fusca* ^37^.

Although different *Sarcophyton* species were subjected to extensive chemical and biological studies^38^, to our knowledge there are no reports regarding the chemical composition of the corals in response to elicitation using large scale untargeted analytical techniques. Metabolomics, defined as the comprehensive profiling of all metabolites within a given sample in a particular physiological state, provide a holistic measure of the global changes occurring post elicitation. Recent developments in analytical techniques and bioinformatics tools^39–41^ have allowed us to monitor metabolites differences among samples in a semi-automated and untargeted manner. Metabolomics makes use mostly of coupled techniques which rely first on chromatographic separation of metabolites followed by mass spectrometry (MS) or nuclear magnetic resonance (NMR) for detection and or structural elucidation of separated peaks. In particular, untargeted ultra-performance liquid chromatography-mass spectrometry (UPLC-MS)-based approach is well suited to reveal the effects of elicitation on samples at metabolite levels^42^. Recently, our group has reported the first comparative metabolomics study of 16 *Sarcophyton* and other soft coral species for investigation of their metabolism in the context of genetic diversity i.e. species type and or growing habitat i.e. aquarium versus sea^38^.

**Figure 1:**
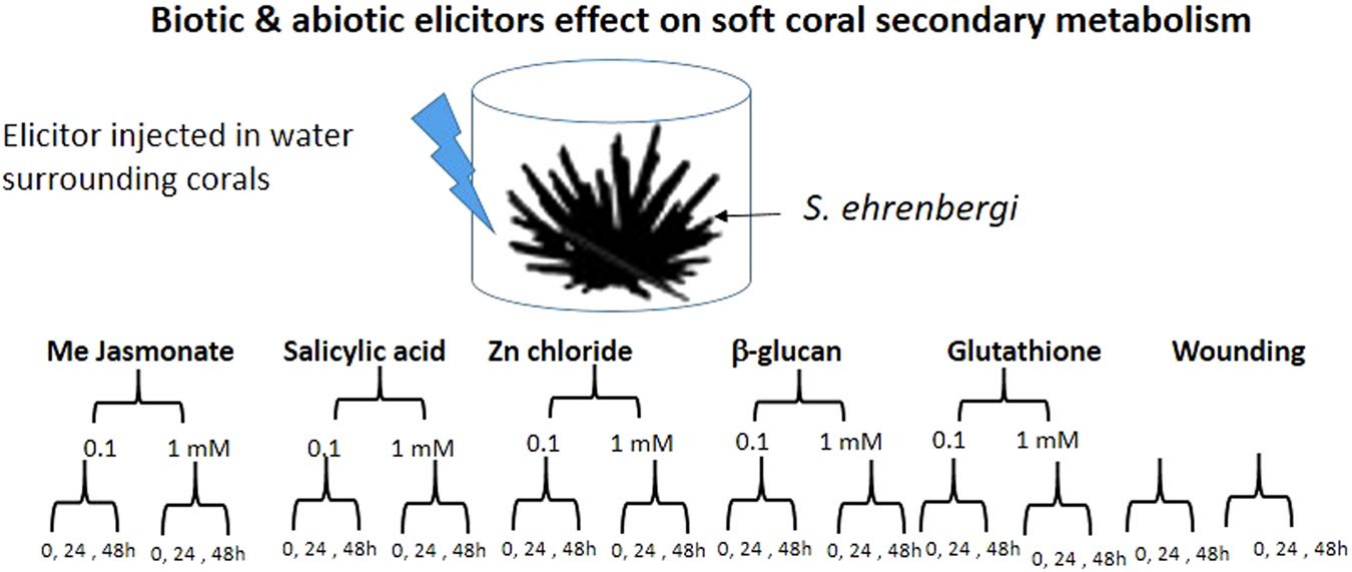
Elicitors application and sampling theme used in this study.

In the present work, a large-scale elicitation experiment was performed using *Sarcophyton ehrenbergi* soft coral and five independent potential chemical elicitors: MeJA (methyl jasmonate), SA (salicylic acid), ZnCl_2_, glutathione and *β*-glucan BG (fungal elicitor), in addition to wounding which can release endogenous elicitors, to provide the first insight into biotic and abiotic stress responses associated with natural product pathways in soft coral with their associated algae (layout of experimental design is shown in Fig. 1). The selection of elicitors was based on previous reports on eliciting animal and plant cell culture system considering that animal coral is a holobiont harboring a zooxanthellae inside. Additionally elicitors were categorized as both biotic i.e., β-glucan and abiotic i.e., heavy metal. The corals and the harboured symbiotic zooxanthellae (algae) were extracted, and analysed using UPLC-MS. Considering the complexity of the dataset and large sample size pool, a total of 72 samples, supervised and unsupervised multivariate data analyses were applied to evaluate the effect of elicitors on coral metabolism and for samples classification in an untargeted manner.

## Results

### Effect of elicitation on photosynthesis efficiency and coral/algal symbiosis

The photosynthesis efficiency of *S. ehrenbergi* with symbiotic zooxanthellae was assessed after treatment with five independent chemical elicitors; *viz*. MeJA, SA, ZnCl_2_, glutathione and *β*-glucan, in addition to wounding which can release an endogenous elicitor signal. No difference in photochemical quantum efficiency was observed in all elicited coral samples, except for samples treated with MeJA which showed slight decrease in comparable pulse amplitude modulated measurements (PAM). The results are presented in (Supplementary Fig. S1).

### Unsupervised multivariate data analyses of *S. ehrenbergi* samples in response to elicitation

**Figure 2:**
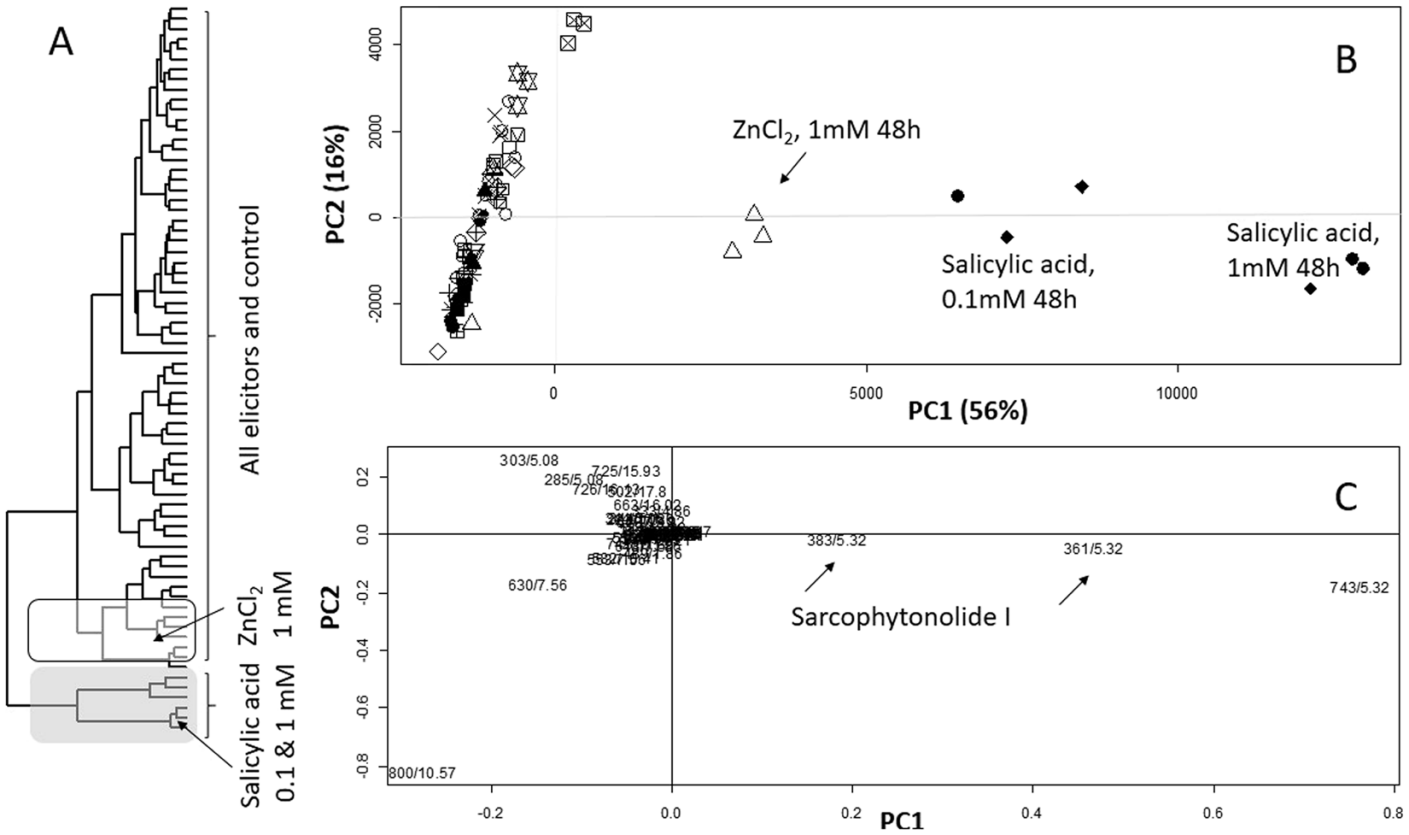
Principal component analysis (PCA) and hierarchical clustering (HCA) of UPLC-MS extracted metabolites from *S*. *ehrenbergi* elicited with methyl jasmonate (MeJA), ZnCl_2_, *β*-glucan, salicylic acid (SA), and glutathione at 0.1 and 1 mM and after wounding, harvested at 0 h (before), 24 h and 48 h post elicitation. (**A**) Score plot of PC1 vs. PC2 scores. (**B**) Loading plot for PC1 & PC2 contributing metabolites and their assignments. The clusters are located at the distinct positions in two-dimensional space described by two vectors of principal component 1 (PC1) = 56% and PC2 = 16%. (**C**) HCA plot. Grey highlighted box in HCA cluster denotes for the cluster most distant from the rest, caused by SA elicitation at 24 h and 48 h.

*Sarcophyton ehrenbergi* samples with symbiotic zooxanthellae (algae) were extracted after elicitation and analysed via UPLC-MS. Some differences in metabolite patterns between *S. ehrenbergi* coral samples were obvious by visual examination of the UPLC-MS chromatograms. Nevertheless, the large dataset generated (72 samples) from this study prompted us to utilize multivariate data analysis *i.e*. principal component analysis (PCA) and hierarchical cluster analysis (HCA) to classify samples and to identify the effect of potential elicitors on the *S. ehrenbergi* metabolome. Principle component analysis is the most used multivariate data analysis model regarded as an unsupervised clustering method that reduces the dimensionality of multivariate data, while preserving most of the variance^43^. A total of 1269 mass signals were extracted from all samples by XCMS software from the UPLC–MS data set acquired in positive ionization mode. The main principal component (PC) to differentiate between samples, i.e. PC1, accounted for 56% of the variance. The score plot revealed that triplicate measurements from the same sample were found to be reproducible, clustering altogether in the score plot. The score plot shows a clear separation between coral samples elicited with 1 mM ZnCl_2_ and 0.1 and 1 mM SA harvested 48 h post elicitation, positioned to the right side along PC1 (positive PC1 values) (Fig. 2A). These were well separated from all other elicited coral samples and control groups located to the left along PC1 (negative PC1 values). It should be noted that no differentiation could be observed along PC2 between coral samples elicited with different concentrations of SA *viz*. 0.1 and 1 mM. The clustering observed in the PCA score plot can be explained in terms of the identified metabolites as revealed from the loading plot (Fig. 2B). Examination of the loading plot indicated that the distant clustering of ZnCl_2_ and SA elicited coral samples was almost exclusively due to mass signals *m/z* 361, 383 and 743 eluted at retention time (*R*_*t*_) 5.32 corresponding to sarcophytonolide I and assigned as [M + H]^+^, [M + Na]^+^ and [2 M + Na]^+^, respectively. Details on metabolites identification strategy has previously been published^38^. Hierarchical cluster analysis, similar to PCA, is an unsupervised data analysis method that establishes relationships among similar groups of samples allowing for analysis of the results in a fairly easier way. The HCA dendrogram showed similar results to those of PCA with clear clustering of *Sarcophyton* samples elicited with 1 mM ZnCl_2_ and 0.1 and 1 mM SA collected 48 h post elicitation (Fig. 2C). This was evident by being clustered most distant from all other elicited coral samples and control groups. Similarly such clustering pattern, especially for SA elicited samples, was mainly attributed to MS signals of sarcophytonolide I, *m/*z 361, as revealed from a clustering heat map plot (Supplementary Fig. S2). The effect of increased production of sarcophytonolide I was clearly apparent in the comparative, reconstructed ion chromatograms for its *m/z* 361 comparing SA (Supplementary Fig. S3A) and ZnCl_2_ (Supplementary Fig. S3B) elicited *Sarcophyton* soft coral samples showing enriched peaks at *R*_*t*_ 5.32 min versus non-elicited control sample (Supplementary Fig. S3C, compare background signal levels of **C** vs. **B** and **A**). It should be noted that no change was observed in major sesequiterpene peaks *viz*. guaiacophine and calamanene^38^ in response to any of the treatment suggesting that for no obvious elicitation effect on corals sesquiterpenes pool.

### Supervised multivariate data analysis of *S. ehrenbergi* samples in response to elicitation

**Figure 4:**
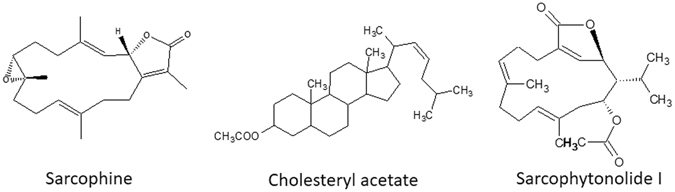
Structures of diterpenes and a sterol showing a marked differential response to SA and ZnCl_2_ elicitation in *S*. *ehrenbergi*.

Having established that elicitation of *S. ehrenbergi* soft corals with SA and ZnCl_2_ led to the accumulation of the diterpenoid sarcophytonolide I, orthogonal projection to latent structures-discriminant analysis (OPLS-DA) was attempted to compare with the elicitor effect interpretation provided by unsupervised PCA and HCA modelling, and to differentiate between ZnCl_2_ and SA elicited coral samples and non-elicited controls. OPLS-DA is a supervised data analysis method, which analyses data according to a *priori* class information assigned to samples before the analysis; making it a sensitive method for extraction of information on changes/differences in the metabolite composition among sample classes. Such modelling enables the identification of the most relevant variables responsible for the discrimination between two predefined sample groups^44^. Soft coral samples elicited with 1 mM ZnCl_2_ harvested at 24 h and 48 h post elicitation was modelled against control samples using OPLS-DA. The derived score plot showed a clear separation between both samples with a total variance (R^2^ = 0.96) and prediction goodness parameter (Q^2^ = 0.99) (Fig. 3A). The S-plot obtained by the OPLS-DA model was used to identify possible biomarkers responsible for the separation observed in the score plot at a cut-off value of P < 0.05 (Fig. 3B). The S-plot results show that ZnCl_2_ elicited coral samples were enriched in the acetylated diterpene “sarcophytonolide I” (*m/z*/*R*_*t*_(*min*) = 743/5.32), as well as a mass signal *m/z*/*R*_*t*_ (*min*) = 429/13.78 corresponding to cholesteryl acetate. In contrast to sarcophytonolide and cholesteryl acetate, elicited coral samples showed a decrease of the abundant C_20_-diterpene(s) sarcophine/*ent*-sarcophine, with an indicative *m/z*/*R*_*t*_ at 317/3.17. The score plot of the OPLS model derived from modelling SA elicited coral samples at 24 h and 48 h versus non-elicited control samples again displays similar trends to those obtained from PCA (Fig. 3C). The S-plot shows SA elicited coral samples to be enriched in sarcophytonolide I (Fig. 3D). The structures of terpenes and a sterol showing differential response to ZnCl_2_ and SA elicitation in *S*. *ehrenbergi* are depicted in Fig. 4. Interestingly, no change was observed in total sesquiterpene content in response to either elicitors.

**Figue 3:**
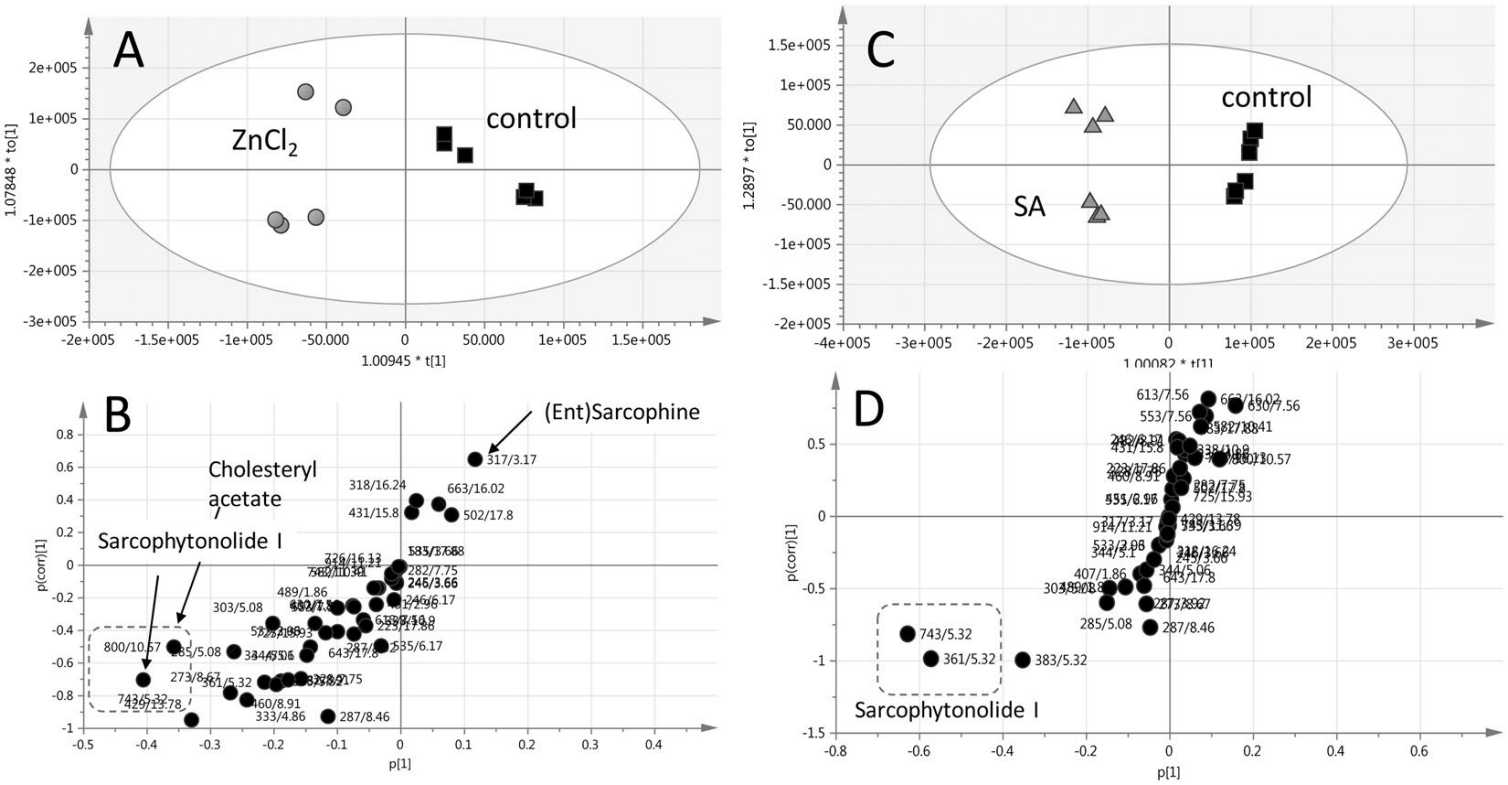
(**A**) OPLS-DA score plots derived from ZnCl_2_ (**A**) and salicylic acid (SA) (**C**) elicitation experiments, each modelled against control. The S-plots from ZnCl_2_ (**B**) and salicylic acid (**D**) show the covariance p[1] against the correlation p(cor) [1] of the variables of the discriminating component of the OPLS-DA model. Cut-off values of P < 0.05 were used; selected variables are highlighted in the S-plot with *m/z* and retention time in minutes and identifications are discussed in the text.

### Quantification of metabolites in elicited soft coral samples

**Figure 5:**
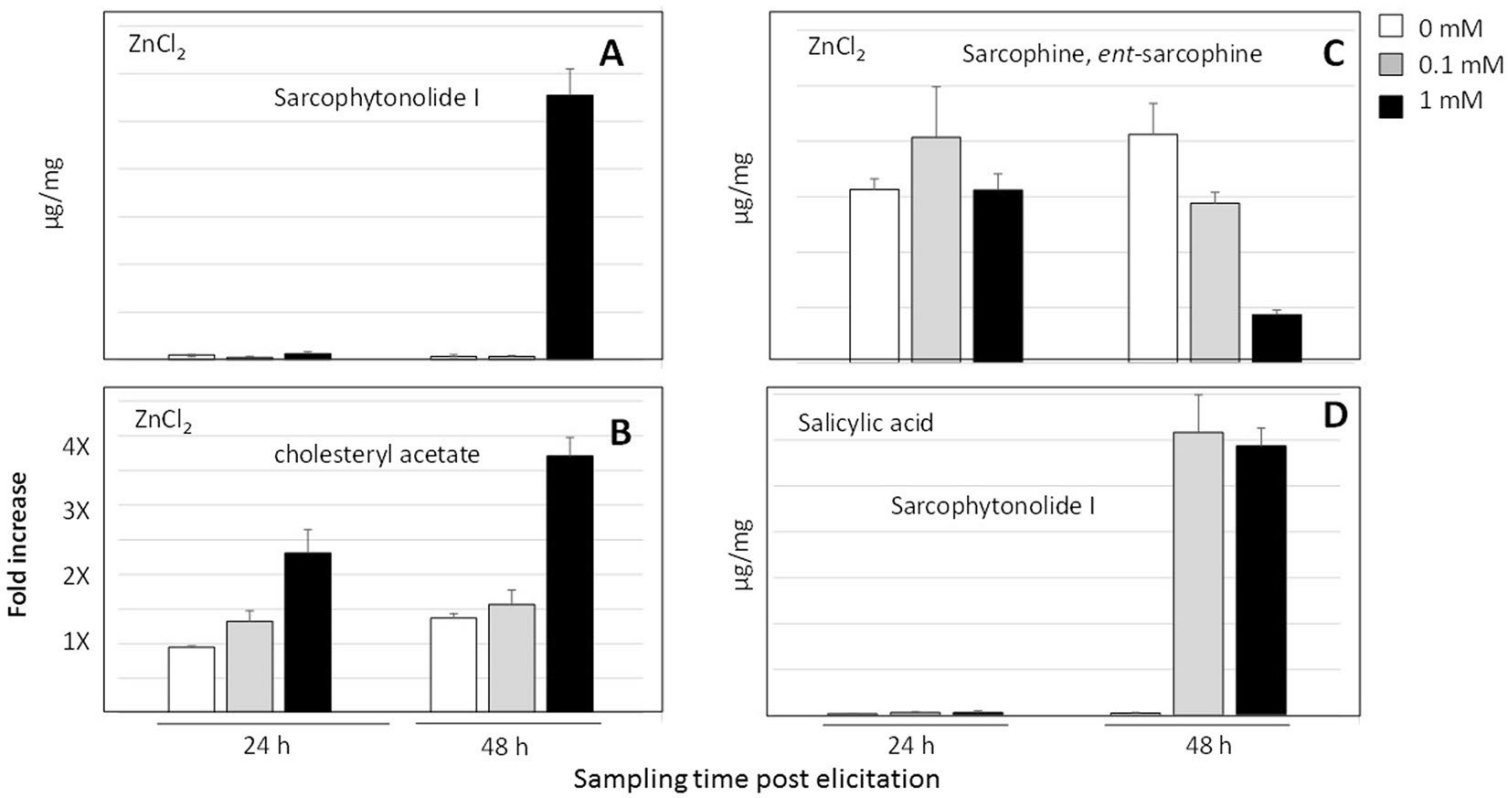
Absolute quantification of major terpenoids and sterols in *S*. *ehrenbergi* soft corals 24 h and 48 h post elicitation. Sarcophytonolide I (**A**), cholesteryl acetate (**B**), sarcophine/*ent*-sarcophine (**C**) contents after ZnCl_2_ elicitation; and Sarcophytonolide I content (**D**) in SA elicited corals.

Absolute quantification of sarcophytonolide I, cholesteryl acetate and sarcophine/*ent*-sarcophine, the major metabolites in ZnCl_2_ and SA elicited *S*. *ehrenbergi* soft corals collected 24 and 48 h post elicitation is shown in (Fig. 5). In agreement with multivariate data analysis results, elicitation with 1 mM ZnCl_2_ led to a 17-fold increase in sarcophytonolide I content (Fig. 5A) reaching a maximum level of 11.5 µg/mg dry coral weight and *ca*. 3-fold increase in cholesteryl acetate level (Fig. 5B). Simultaneously, a 4.7-fold decrease occurred for sarcophine/*ent*-sarcophine to reach a level of 1.3 µg/mg (Fig. 5C). Sarcophytonolide I content showed the most remarkable 132 and 126-fold increase with 0.1 and 1 mM SA and was induced to maximum values of 92.4 and 88 µg/mg at 48 h post elicitation, respectively (Fig. 5D). Interestingly, with ZnCl_2_ or SA elicitation, significant effects became evident only after 48 h for the decrease in sarcophine/*ent*-sarcophine and increase in sarcophytonolide I (Fig. 5A,C,D), while the membrane compound cholesteryl acetate responded more immediately (Fig. 5B).

### The effect of elicitation on soft coral harboured zooxanthellae (Symbiodinium) enriched extracts

**Figure 6:**
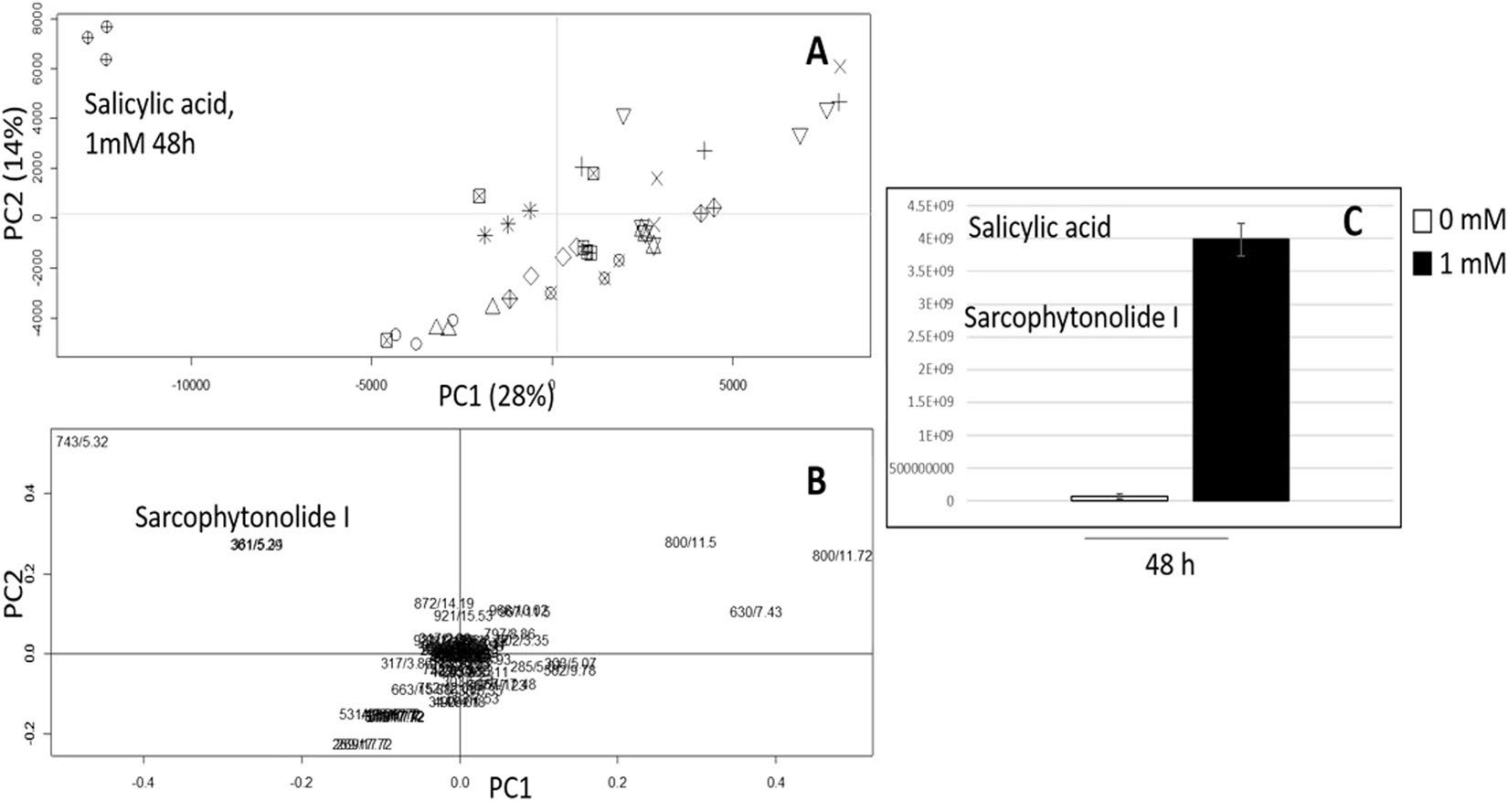
Principal component analysis (PCA) of UPLC-MS of zooxanthellae enriched extracts after elicitation with 1 mM MeJA, ZnCl_2_, *β*-glucan, salicylic acid, glutathione, and wounding, harvested at 0 h (before), 24 h and 48 h post elicitation. (**A**) Score plot of PC1 vs. PC2 scores. (**B**) Loading plot for PC1 & PC2 contributing metabolites and their assignments. The clusters are located at the distinct positions in two-dimensional space described by two vectors of principal component 1 (PC1) = 28% and PC2 = 14%. Quantification of sarcophytonolide I (**C**) in zooxanthellae enriched extracts of salicylic acid elicited coral samples at 48 h post elicitation.

Zooxanthellae harboured within *S*. *ehrenbergi* soft coral tissues were isolated from elicited coral samples in an attempt to determine the impact of elicitation on the different metabolites. Phylogenetic analysis of the harboured zooxanthellae was carried out using internal transcribed spacer of ribosomal DNA (ITS-rDNA) sequence and found to match most closely with *Symbiodinium sp*. AB591706.1 isolated from a *Sarcophyton sp*. from Solomon island with 99% homology (data from NCBI GenBank). Both zooxanthellae-enriched elicited and control samples were extracted for metabolites analysis using UPLC-MS under the same conditions used for coral tissues then further evaluated using PCA for sample classification. Consistent with soft coral samples, the score plot showed distant separation of samples elicited with 1 mM SA collected at 48 h post elicitation with negative PC1 score values (Fig. 6A), while examination of the loading plot revealed that mass signals, *m/z* 361 and 743 were the discriminatory signals corresponding to sarcophytonolide I (Fig. 6B). Relative quantification of sarcophytonolide I in zooxanthellae-enriched samples elicited with 1 mM SA at 48 h showed up to 54-fold increase in its content (Fig. 6C). It should be noted that primary metabolites analysis in zooxanthellae fraction revealed for enrichment in glucose reaching 22% of total metabolites composition (A) compared to 3% in coral tissue (B) analysed under same conditions using GC-MS (Supplementary Fig. 4) and confirming that our protocol is efficient to isolate zooxanthellae from corals.

## Discussion

The effects of five potential chemical elicitors (four organic, one inorganic) and physical induction of endogenous elicitors; *viz*. MeJA, SA, glutathione, *β*-glucan, ZnCl_2_, and wounding on the secondary metabolite profile of *Sarcophyton ehrenbergi* coral with harboured zooxanthellae was investigated in the current study. The samples showed comparable pulse amplitude modulated (PAM) measurements suggesting no major effect of elicitation on coral/algal photosynthesis efficiency. Soft corals possess endosymbiotic dinoflagellates of the genus *Symbiodinium* (commonly referred to as zooxanthellae), where the host coral tissue is provided by up to 98% of its metabolic needs by accessing photosynthates and maybe also secondary metabolites produced by zooxanthellae^45^. The absence of a remarkable stress response (except for MeJA) in the corals primary production with most applied potential elicitors is indicative that the chosen ones may either be inactive in *Sarcophyton* (i.e. they are not elicitors), or they provide selective elicitation, e.g. of certain bioactive secondary metabolites, a property that would make the respective elicitors ideal candidates for production of the respective metabolites.

Multivariate data analysis of the metabolites pattern of elicited *S*. *ehrenbergi* soft coral samples showed that SA and ZnCl_2_ were successfully able to induce accumulation of defined terpenoids in soft corals. Sarcophytonolide I was identified as the discriminatory metabolite responsible for elicitation segregation effect. Sarcophytonolide I, is an acetylated cembranoid diterpene having an *α*,*β-*unsaturated butyrolactone moiety, previously isolated from *Sarcophyton latum* ^46^ and identified in several other *Sarcophyton* species. Terpenes are usually acquired or *de novo* biosynthesized by the corals, where they act as chemical defence for the organism against different biotic stressors^47, 48^.

Salicylic acid is a naturally occurring plant hormone and one of the most well characterized stress signaling molecules *in planta*, known to up-regulate defence genes and related enzymes of the secondary metabolic pathway in response to pathogen attack^49^. A previous study reporting the ability of SA to induce the biosynthesis of the diterpene “fuscol” in isolated dinoflagellates of *Eunicea fusca* corals^37^, is in agreement with our current results. Salicylic acid has also been shown to elicit the accumulation of secondary metabolites in several terrestrial plants *viz*. artemisinin in *Artemisia annua* ^50^, triterpene saponin in *Quillaja brasiliensis* ^51^ and glycyrrhizin in *Glycyrrhiza glabra* ^52^ and extends that now to encompass diterpenes in sea corals. Whether SA is involved in corals protection against their predators attack has yet to be examined for both coral aquarium and sea corals.

Metal ions, such as Ca^2+^, Zn^2+^, Cu^2+^ and Ag^+^ ions are abiotic inorganic elicitors, when added in appropriate concentrations, they can act as signalling molecules; triggering secondary metabolites production^23^. Our results for ZnCl_2_ elicitation of diterpenes in *Sarcophyton* coral agree with previous reports in which CdCl_2_ and CuCl_2_ induced accumulation of diterpene lactones in *Andrographis paniculata* suspension culture^53^. While Zn may act as an elicitor, its effects might also be direct, *i*.*e*., not transmitted by elicitation. Since Zn is an important cofactor of enzymes, its addition to unnatural levels, potentially even increased by biotic accumulation, may increase or alter the activity of terpenoid pathway enzymes. It is known that some terpene synthases alter their product spectrum depending on the levels of available transition metals^54^.

In contrast, results of both PCA and HCA analysis demonstrated the failure of MeJA elicitation to increase the diterpene content of *S*. *ehrenbergi*, although several studies report on its ability to induce terpene biosynthesis in both marine and terrestrial organisms^31, 37, 55^. Such result might be attributed to the decrease in photosynthesis efficiency induced by MeJA in soft coral samples at the examined dose. Methyl jasmonate is a well-known inducer of terpenes *in planta* and could inhibit photosynthesis in corals by negatively impacting quantum efficiency of photosystem II. The fact that no increase of secondary metabolite production was observed in the current study might lie in a special control of MeJA levels or effect in harboured algae. Nevertheless, this is just an early hypothesis which would have to be pursued by comparing metabolomic profiles in zooxanthellate versus azooxanthellate corals. Such an approach was applied for identifying symbiosis specific proteins during the natural onset of symbiosis using proteomics^56^. The lack of change in the diterpene content after mechanical wounding is in agreement with previous reports from *Pseudopterogorgia elisabethae* in which artificial wounding failed to increase pseudopterosin diterpene glycoside levels^36^. However, in plants it was shown that the type of wounding can result in different responses, and this needs to be checked in the future^27^. Glutathione and *β*-glucan failed to show any significant increase in *S*. *ehrenbergi* terpenoidal content, i.e. they appear to not be elicitors at all in this species.

Supervised OPLS-DA analysis of 1 mM ZnCl_2_ and SA elicited coral samples were modelled against (non-elicited) control samples. The results showed that soft coral samples elicited with 1 mM ZnCl_2_ harvested at 48 h post elicitation were enriched in the acetylated diterpene, Sarcophytonolide I, in addition to cholesteryl acetate, which already increased after 24 h. At the same time, the content of the otherwise dominant C_20_-diterpene(s) sarcophine/*ent*-sarcophine decreased, i.e. one of the major metabolites identified in several *Sarcophyton* species is depleted^38^. This hints at the possibility that the early precursors of the terpenoid pathway are not upregulated, but the increase of two major products of elicitation are formed at the expense of sarcophines’ biosynthesis from a regulated common precursor.

Salicylic acid elicited coral samples much faster and stronger, giving high (apparently maximum) levels already at 24 h for sarcophytonolide I. The significant increase in the levels of acetylated diterpene and sterol *viz*., sarcophytonolide and cholesteryl acetate, suggest an activation of specific acetyl transferases as part of the defence response to ZnCl_2_ and SA. This hypothesis is supported by previous findings in which elicitation of *Taxus cuspidata* with SA was found to increase the content of 10-deacetylbaccatin III-10-*O*-acetyltransferase (10-DBAT); an enzyme that catalyses the formation of baccatin II, and ultimately promoting the production of taxol^57^. The relative change in sarcophytonolide I and cholesteryl acetate levels, presumably on the expense of other terpenoids, negates a *de novo* biosynthetic activation in response to elicitation but is likely to involve downstream acetylation reaction activation. In brief, these results suggest that SA elicitation is superior to ZnCl_2_ in eliciting the production of sarcophytonolide I without affecting sarcophine content, a major diterpene in corals, ascribed with potent anti-cancer effects^58^.

The analysis of zooxanthellae enriched extracts showed similar results to those obtained from soft corals. Whether the increased diterpene is biosynthesized inside corals and then transported to zooxanthellae or made wholly or in part by this symbiont has yet to be examined. Generally *in planta*, diterpenes are biosynthesized *via* the non-mevalonic (deoxyxylulose phosphate) pathway, whereas sterols i.e. cholesterol are formed via the mevalonate pathway^59^. To confirm whether such ascenario occurs in soft corals, acetyl coA isotopomers feeding experiment^60^ or the addition of terpenoid pathway inhibitors^61^ could provide insight into the diterpene biosynthesis in corals. It should be noted that in corals, several factors complicate such studies considering that corals harbour several other organisms including zooxanthellae and micro- organisms that are likely to contribute to the secondary metabolic pathways^1^. Many bacteria and plant chloroplasts utilize the mevalonate-independent deoxyxylulose phosphate (DXP) pathway to produce terpenoids^62^. The study of symbiotic relationships in soft corals is indeed multidisciplinary and should involve collaborative research in coral phylogeny, physiology, and biochemistry.

The combination of metabolomic techniques with multivariate data analysis methods is a reliable and well-accepted investigative tool that clearly demonstrates the changes that occurred in the soft corals. Our results show that manipulation of the terpenoid content of *S*. *ehrenbergi* soft coral is feasible, with both quantitative and qualitative changes occurring in response to SA and abiotic ZnCl_2_ elicitation. The present work provides the first metabolic evidence for a possible activation/expression of specific acetyltransferases in response to SA or ZnCl_2_ elicitation, leading to an increase in acetylated diterpene content. Sarcophytonolide I was the prominent discriminatory biomarker in elicited coral/algal extracts and algal enriched extracts as well. In addition, the data reveal a broader effect of ZnCl_2_, being elicitor or cofactor based, on secondary metabolic pathway changes in corals affecting sterols and diterpenes. In future work, monitoring enzymatic activity or gene expression levels related to the biosynthesis of these diterpenes might provide a deeper understanding of its regulation in *Sarcophyton* soft corals in response to elicitation. Moreover, results are not decisive with regards to the biogenetic source of metabolites whether from coral polyps or their inside symbionts. Further studies should be pursued with other biotic and abiotic elicitors on other coral species to provide more evidence for the role of terpenes in coral defence response and whether such induction response for SA is unique for *Sarcophyton* species or more general in other soft corals.

## Methods

### Soft coral material

The soft coral *Sarcophyton* of the family Alcyoniidae is the most common genus along several sea coasts *S*. *ehrenbergi* soft corals were propagated in the aquarium facility of the Leibniz Centre for Tropical Marine Ecology, Bremen, Germany. Corals were kept at 26 °C in a 2500 L recirculation system with 50 cm distance between coral nubbins and a blue/white combination of two 39 W fluorescence light bulbs^38^.

### Chemicals and reagents

Solvents used were (LC–MS grade) grade purchased from Baker (The Netherlands), milliQ water was used for LC analysis. Sarcophine standard was purchased from AG Scientific, San Diego, CA (St. Louis, MO, USA). Chromoband C18 (500 mg, 3 mL) cartridge from Macherey and Nagel (Düren, Germany). All biotic and abiotic elicitors were purchased from Sigma Aldrich (St. Louis, MO, USA).

### Elicitation

For elicitation, corals were placed in small glass jars of an average volume of 600 mL with a close lid through which a teflon tube provided aeration to corals. Elicitors were prepared in deionized water at a stock solution of 100 mM and further diluted in coral glass tanks to a concentration of 0.1 and 1 mM. Wounding was made by making two cuts into top coral heads i.e., polyp zone, of *ca*. 2 cm length and 0.5 cm depth using sterile scalpels. The remaining flask was used as control by the addition of the same volume of sterile deionized water kept at 28 ± 1 °C, with a 12 h photoperiod. Corals were harvested at zero time and after 24 h and 48 h post elicitation, and the harvested corals were kept at −80 °C until further analysis. Corals were treated with each elicitor in triplicates by eliciting three independent corals placed in three different glass jars (Supplementary Fig. S1).

### Pulse amplitude modulation (PAM) fluorometry measurements

Photosystem II (PSII) photochemical efficiency was measured with a PAM-2500 portable chlorophyll fluorimeter (Walz, Germany) on three random nubbins per coral fragment. The steady state (effective) quantum yield of chlorophyll fluorescence, Y(II), in PSII, was calculated as Fm′-Fo′/Fm′, where Fo′, Fm′ are the steady-state and maximum yields of chlorophyll fluorescence measured under ambient light, respectively^63^.

### Soft coral extraction procedure and sample preparation for UPLC-MS analyses

Soft coral extraction followed the protocol described in a previous report^38^ with few modifications. Approximately 20 mg tissue from powdered freeze-dried soft coral tissues were ground with a pestle in a mortar under liquid nitrogen. The powder was then homogenized with 1.0 mL 100% ethanol containing 5 µg/ml umbelliferone (as internal standard for UPLC-MS) using an ultrasonic bath for 20 min. Extracts were then centrifuged at 12000 g for 5 min to remove debris. For UPLC-MS analyses, 500 µL were aliquoted and filtered through 22 µm filter. A volume of 3 µL were used for UPLC-MS analysis. For each specimen, 3 biological replicates were extracted and analysed in parallel under the same conditions.

### Zooxanthellae samples preparation for UPLC-MS analysis

Zooxanthellae harboured within the coral tissues were isolated by scrapping the coral head using a liquid nitrogen dipped blade. The scraped-off liquid was collected and lyophilized and the dried pellet was extracted as for corals. 10 mg of these powdered freeze-dried zooxanthellae were homogenized with 1.0 mL 100% ethanol containing 5 µg/mL umbelliferone using an ultrasonic bath for 20 min. The zooxanthellae extracts were then centrifuged at 12000 g for 5 min to remove debris, and filtered through 22 µm filter. The filtrate was diluted with a three-fold volume of 100% ethanol and then used for UPLC-MS analysis. For each specimen, 3 biological replicates were extracted and analysed in parallel under the same conditions.

### DNA extraction and PCR analyses of ITS-rDNA: *Symbiodinium* (zooxanthellae)

To identify *Symbiodinium* (zooxanthellae) species harboured inside *Sarcophyton* coral, gene sequences analysis was attempted following a previously described protocol^64^. Briefly, soft coral head was cut into small pieces of approximately 20 mg, and treated with 20 mL proteinase K in 180 mL ALT buffer for 4–6 h at 56 °C. Then, total genomic DNA was extracted from each specimen using a spin-column DNeasy Animal DNA Extraction kit following the manufacturer’s protocol (QIAGEN, Tokyo, Japan). The internal transcribed spacer of ribosomal DNA (ITS-rDNA) was amplified using zooxanthellae-specific zITSf (5′-CCGGTGAATTATTCGGACTGACGCAGT-3′). The purified PCR-amplified DNA fragments were cloned into the pCR2.1 vector of the TOPO TA Cloning Kit (Invitrogen, Carlsbad, CA, USA) and sequenced using an ABI PRISM Big Dye Terminator cycle sequencing kit Ver. 3.1 (Applied Biosystems, Foster City, CA).

### UPLC-PDA-MS analysis

High resolution UPLC-MS analysis conditions adopted were previously described^38^. Chromatographic separation was performed on an Acquity UPLC system (Waters) equipped with a HSS T3 column (100 × 1.0 mm, particle size 1.8 µm; Waters). The analysis was carried out using a binary gradient elution system at a flow rate of 150 µL min^−1^: 0 to 1 min, isocratic 95% A (water/formic acid, 99.9/0.1 [v/v]), 5% B (acetonitrile/formic acid, 99.9/0.1 [v/v]); 1 to 16 min, linear from 5 to 95% B; 16 to 18 min then isocratic 95% B; 18 to 20 min, and finally, isocratic 5% B. Detection range of eluted compounds was from *m/z* 100 to 1000 in positive ion mode, obtained using the following instrument settings: nebulizer gas, nitrogen, 1.6 bar; dry gas, nitrogen, 6 liters min^−1^, 190 °C; capillary, −5500 V; in-source CID energy, 0 V; hexapole RF, 100 Vpp; quadrupole ion energy, 5 eV; collision gas, argon; collision energy, 10 eV; collision RF 200/400 Vpp (timing 50/50); transfer time, 70 µs; prepulse storage, 5 µs; pulser frequency, 10 kHz; spectra rate, 3 Hz.

### MS data processing for multivariate analysis: PCA, HCA and OPLS

Relative quantification and comparison of *Sarcophyton* and their harboured zooxanthellae metabolic profiles after UPLC-MS was performed using XCMS data analysis software under R 2.9.2 environment, which can be downloaded for free as an R package from the Metlin Metabolite Database^65^. Native UPLC-MS files from Xcalibur 1.4 (Thermo Fisher Scientific, Inc., Waltham, MA) were firstly converted into netCDF files using the File Converter tool. Files were arranged in one folder that was set as the file source. Peaks were subsequently extracted using XCMS under R 2.9.2 environment with signal-to-noise ratio set at 4. After peak extraction and grouping, nonlinear retention time correction of peaks was accomplished in two iterative cycles with descending bandwidth (bw). This was accomplished by manually decreasing the bw parameter (from 5 to 2 s) with a signal to noise set at 3. The resulting peak list was further processed using the Microsoft Excel software (Microsoft, Redmond, WA), where the ion features were normalized to the total integrated area (1,000) per sample and then Pareto scaled prior to principal component analysis. This provides similar weights for all the variables. PCA was then performed on the MS-scaled data to visualize general clustering, trends, and outliers among all samples on the scores plot. Script used for UPLC-MS files data processing using XCMS is detailed for in a “supplementary text material”. R packages (PCA methods & Heatplus), were used for PCA & HCA analyses. The software can be downloaded freely as an R package from the Metlin Metabolite Database under R 2.9.2 environment. OPLS-DA was performed with the program SIMCA-P Version 13.0 (Umetrics, Umeå, Sweden).

### Absolute quantification of metabolites via UPLC-MS

Sarcophytonolide I, cholesteryl acetate and sarcophine/*ent*-sarcophine were quantified from the calibration curves of sarcophine, detected using mass spectrometer. Standard calibration curves were conducted for the standard prepared at 1, 10, 100 and 1000 µg/ml and used for absolute quantification of targeted diterpenes. Quantification assays were carried out in triplicates. Recovery percentages for sarcohpine added prior to the extraction of coral samples was 92% with a limit of detection (LOD) and limit of quantification (LOQ) of 1.02 and 3.42 µg/ml, respectively.

### GC-MS analysis of silylated primary metabolites

For analysis of primary metabolites (*viz*. amino acids, organicacids, and sugars), 100 μL of 50% aqueous extract (prepared by extracting 100 mg of zooxanthellae or coral tissue in 5 ml 50% MeOH with sonication for 30 min followed by centrifugation) was evaporated under nitrogen till dryness. For derivatization, 150 μL of N-methyl- N-(trimethylsilyl)-trifluoroacetamide (MSTFA) was then added and incubated at 60 °C for 45 min. The samples were equilibrated at 28 °C and subsequently analyzed using GCMS. Silylated derivatives were separated on a Rtx-5MS (30 m length, 0.25 mm inner diameter, and 0.25 μm film) column. Injections were made in a (1:1) split mode and the GC was operated under the following conditions: injector 280 °C, column oven 80 °C for 2 min, then programmed at a rate of 5 °C/min to 315 °C, kept at 315 °C for 12 min. He carrier gas at 1 mL min^−1^. The transfer line and ion–source temperatures were adjusted at 280 and 180 °C, respectively. The mass spectrometer was operated in the electron ionization mode at 70 eV. The scan range was set at 50–650 *m/z*.

## Electronic supplementary material

Supplementary Information

## References

[1] Changyun W (2008). Chemical defensive substances of soft corals and gorgonians. Acta Ecol. Sin..

[2] Rocha J, Peixe L, Gomes N, Calado R (2011). Cnidarians as a source of new marine bioactive compounds—An overview of the last decade and future steps for bioprospecting. Mar drugs.

[3] Coll JC (1992). The chemistry and chemical ecology of octocorals (Coelenterata, Anthozoa, Octocorallia). Chem. Rev..

[4] Haverkort-Yeh RD (2013). A taxonomic survey of Saudi Arabian Red Sea octocorals (Cnidaria: Alcyonacea). Mar Biodivers.

[5] Fabricius K (1997). Soft coral abundance on the central Great Barrier Reef: effects of Acanthaster planci, space availability, and aspects of the physical environment. Coral Reefs.

[6] Blunt JW, Copp BR, Keyzers RA, Munro M, Prinsep MR (2013). Marine natural products. Nat. Prod. Rep.

[7] Blunt JW, Copp BR, Keyzers RA, Munro MH, Prinsep MR (2015). Marine natural products. Nat. Prod. Rep..

[8] Elkhateeb A (2014). New terpenes from the Egyptian soft coral Sarcophyton ehrenbergi. Mar Drugs.

[9] Lin W-Y (2014). Bioactive Cembranoids, Sarcocrassocolides P–R, from the Dongsha Atoll Soft Coral Sarcophyton crassocaule. Mar Drugs.

[10] Hegazy MEF (2015). Molecular Architecture and Biomedical Leads of Terpenes from Red Sea Marine Invertebrates. Mar Drugs.

[11] Yang B (2012). Cembrane diterpenes chemistry and biological properties. Curr Org Chem.

[12] Hegazy M-EF (2012). Bioactive hydroperoxyl cembranoids from the Red Sea soft coral Sarcophyton glaucum. Mar Drugs.

[13] Lin W-Y (2010). Cytotoxic and anti-inflammatory cembranoids from the Dongsha Atoll soft coral Sarcophyton crassocaule. Bioorg. Med. Chem..

[14] González, Y., Torres-Mendoza, D., Jones, G. E. & Fernandez, P. L. Marine Diterpenoids as Potential Anti-Inflammatory Agents. *Mediators*. *Inflamm*. **2015** (2015). 10.1155/2015/263543 PMC4619941 26538822

[15] Ferchmin P (2009). Actions of octocoral and tobacco cembranoids on nicotinic receptors. Toxicon.

[16] Al-Footy KO, Alarif WM, Asiri F, Aly MM, Ayyad S-EN (2015). Rare pyrane-based cembranoids from the Red Sea soft coral Sarcophyton trocheliophorum as potential antimicrobial–antitumor agents. Med. Chem. Res..

[17] Cheng S-Y (2010). Cembranoids from the octocoral Sarcophyton ehrenbergi. J. Nat. Prod..

[18] Fahmy H, Khalifa SI, Konoshima T, Zjawiony JK (2004). An improved synthesis of 7, 8-epoxy-1, 3, 11-cembratriene-15R (α), 16-diol, a cembranoid of marine origin with a potent cancer chemopreventive activity. Mar Drugs.

[19] Badria FA (1998). Sarcophytolide: a new neuroprotective compound from the soft coral Sarcophyton glaucum. Toxicology.

[20] Leal MC, Calado R, Sheridan C, Alimonti A, Osinga R (2013). Coral aquaculture to support drug discovery. Trends Biotechnol..

[21] Khalesi, M. K., Beeftink, R. & Wijffels, R. The soft coral Sinularia flexibilis: potential for drug development in *Advances in Coral Husbandry in Public Aquariums* Vol. 2 (eds Leewis, R. J. & Janse, M.) 47-60 (Burgers’ Zoo, Arnhem, The Netherlands, 2008).

[22] Sipkema D (2005). Large‐scale production of pharmaceuticals by marine sponges: Sea, cell, or synthesis?. Biotechnol. Bioeng..

[23] Radman R, Saez T, Bucke C, Keshavarz T (2003). Elicitation of plants and microbial cell systems. Biotechnol. Appl. Biochem..

[24] Zhong J-J (2002). Plant cell culture for production of paclitaxel and other taxanes. J. Biosci. Bioeng..

[25] Wang, J. W. & Wu, J. Y. Effective elicitors and process strategies for enhancement of secondary metabolite production in hairy root cultures in *Biotechnology of Hairy Root Systems* 55–89 (Springer, 2013). 10.1007/10_2013_183 23467807

[26] Namdeo A (2007). Plant cell elicitation for production of secondary metabolites: a review. Pharmacogn. Rev..

[27] Mithofer A, Wanner G, Boland W (2005). Effects of feeding Spodoptera littoralis on lima bean leaves. II. Continuous mechanical wounding resembling insect feeding is sufficient to elicit herbivory-related volatile emission. Plant Physiol..

[28] Wang H (2010). Effects of exogenous methyl jasmonate on artemisinin biosynthesis and secondary metabolites in Artemisia annua L. Ind. Crops. Prod..

[29] Ram M, Prasad K, Singh S, Hada B, Kumar S (2013). Influence of salicylic acid and methyl jasmonate elicitation on anthocyanin production in callus cultures of Rosa hybrida L. Plant Cell Tissue Organ Cult..

[30] Wang J, Qian J, Yao L, Lu Y (2015). Enhanced production of flavonoids by methyl jasmonate elicitation in cell suspension culture of Hypericum perforatum. Bioresour Bioprocessing.

[31] Wasternack C, Hause B (2013). Jasmonates: biosynthesis, perception, signal transduction and action in plant stress response, growth and development. An update to the 2007 review in Annals of Botany. Ann. Bot..

[32] Yukimune Y, Tabata H, Higashi Y, Hara Y (1996). Methyl jasmonate-induced overproduction of paclitaxel and baccatin III in Taxus cell suspension cultures. Nat. Biotechnol..

[33] Wang C, Wu J, Mei X (2001). Enhancement of taxol production and excretion in Taxus chinensis cell culture by fungal elicitation and medium renewal. Appl. Microbiol. Biotechnol..

[34] Martin D, Tholl D, Gershenzon J, Bohlmann J (2002). Methyl jasmonate induces traumatic resin ducts, terpenoid resin biosynthesis, and terpenoid accumulation in developing xylem of Norway spruce stems. Plant Physiol..

[35] Zhao J-L, Zhou L-G, Wu J-Y (2010). Effects of biotic and abiotic elicitors on cell growth and tanshinone accumulation in Salvia miltiorrhiza cell cultures. Appl. Microbiol. Biotechnol..

[36] Thornton RS, Kerr RG (2002). Induction of pseudopterosin biosynthesis in the gorgonian Pseudopterogorgia elisabethae. J. Chem. Ecol..

[37] Newberger NC, Ranzer LK, Boehnlein JM, Kerr RG (2006). Induction of terpene biosynthesis in dinoflagellate symbionts of Caribbean gorgonians. Phytochemistry.

[38] Farag MA (2016). Soft Corals Biodiversity in the Egyptian Red Sea: A Comparative MS and NMR Metabolomics Approach of Wild and Aquarium Grown Species. J. Proteome Res..

[39] Kim HK, Choi YH, Verpoorte R (2011). NMR-based plant metabolomics: where do we stand, where do we go?. Trends Biotechnol..

[40] Sumner LW, Lei Z, Nikolau BJ, Saito K (2015). Modern plant metabolomics: advanced natural product gene discoveries, improved technologies, and future prospects. Nat. Prod. Rep.

[41] Saccenti E, Hoefsloot HC, Smilde AK, Westerhuis JA, Hendriks MM (2014). Reflections on univariate and multivariate analysis of metabolomics data. Metabolomics.

[42] Farag MA, El Sayed AM, El Banna A, Ruehmann S (2015). Metabolomics reveals distinct methylation reaction in MeJA elicited Nigella sativa callus via UPLC–MS and chemometrics. Plant Cell Tissue Organ Cult..

[43] Allwood JW, Ellis DI, Goodacre R (2008). Metabolomic technologies and their application to the study of plants and plant–host interactions. Physiol. Plant..

[44] Bylesjö M (2006). OPLS discriminant analysis: combining the strengths of PLS-DA and SIMCA classification. J. Chemom.

[45] Sammarco PW, Strychar KB (2013). Responses to high seawater temperatures in zooxanthellate octocorals. PloS one.

[46] Yan XH, Li ZY, Guo YW (2007). Further new cembranoid diterpenes from the Hainan soft coral Sarcophyton latum. Helv. Chim. Acta.

[47] Gross H, König GM (2006). Terpenoids from marine organisms: unique structures and their pharmacological potential. Phytochem Rev..

[48] Gershenzon J, Dudareva N (2007). The function of terpene natural products in the natural world. Nat. Chem. Biol..

[49] Angelova Z, Georgiev S, Roos W (2006). Elicitation of plants. Biotechnol. Biotechnol. Equip..

[50] Pu G-B (2009). Salicylic acid activates artemisinin biosynthesis in Artemisia annua L. Plant Cell Rep..

[51] de Costa F, Yendo ACA, Fleck JD, Gosmann G, Fett-Neto AG (2013). Accumulation of a bioactive triterpene saponin fraction of Quillaja brasiliensis leaves is associated with abiotic and biotic stresses. Plant Physiol. Biochem..

[52] Shabani L, Ehsanpour A, Asghari G, Emami J (2009). Glycyrrhizin production by *in vitro* cultured Glycyrrhiza glabra elicited by methyl jasmonate and salicylic acid. Russ. J. Plant Physiol..

[53] Gandi S, Rao K, Chodisetti B, Giri A (2012). Elicitation of andrographolide in the suspension cultures of Andrographis paniculata. Appl. Biochem. Biotechnol..

[54] Frick S (2013). Metal ions control product specificity of isoprenyl diphosphate synthases in the insect terpenoid pathway. Proceedings of the National Academy of Sciences of the United States of America.

[55] Laskaris G (1999). Induction of geranylgeranyl diphosphate synthase activity and taxane accumulation in Taxus baccata cell cultures after elicitation by methyl jasmonate. Plant Sci.

[56] Barneah O, Benayahu Y, Weis V (2006). Comparative proteomics of symbiotic and aposymbiotic juvenile soft corals. Mar. Biotechnol..

[57] Zhang JF, Gong S, Guo ZG (2011). Effects of different elicitors on 10‐deacetylbaccatin III‐10‐O‐acetyltransferase activity and cytochrome P450 monooxygenase content in suspension cultures of Taxus cuspidata cells. Cell Biol Int Rep.

[58] Arif JM (2007). Antiproliferative potential of sarcophine and its semisynthetic sulfur-containing derivatives against human mammary carcinoma cell lines. J Nat Med.

[59] Dewick PM (2002). The biosynthesis of C 5–C 25 terpenoid compounds. Nat. Prod. Rep.

[60] Schuhr CA (2003). Quantitative assessment of crosstalk between the two isoprenoid biosynthesis pathways in plants by NMR spectroscopy. Phytochem Rev..

[61] Kuzuyama T, Shimizu T, Takahashi S, Seto H (1998). Fosmidomycin, a specific inhibitor of 1-deoxy-D-xylulose 5-phosphate reductoisomerase in the nonmevalonate pathway for terpenoid biosynthesis. Tetrahedron Lett..

[62] Rohmer M (1999). The discovery of a mevalonate-independent pathway for isoprenoid biosynthesis in bacteria, algae and higher plants†. Nat. Prod. Rep.

[63] Lichtenthaler H, Buschmann C, Knapp M (2005). How to correctly determine the different chlorophyll fluorescence parameters and the chlorophyll fluorescence decrease ratio RFd of leaves with the PAM fluorometer. Photosynthetica.

[64] Aratake S (2012). Soft coral Sarcophyton (Cnidaria: Anthozoa: Octocorallia) species diversity and chemotypes. PLoS One.

[65] Smith CA, Want EJ, O’Maille G, Abagyan R, Siuzdak G (2006). XCMS: processing mass spectrometry data for metabolite profiling using nonlinear peak alignment, matching, and identification. Anal. Chem..

