# Supplementary material for: Metabolomics reveals biotic and abiotic elicitor effects on the soft coral Sarcophyton ehrenbergi terpenoid content
Source: Sci Rep. 2017 Apr 5;7:648. doi: 10.1038/s41598-017-00527-8 (PMC5428729; doi:10.1038/s41598-017-00527-8)
Supplement: Supplementary file 1 — Supplementary Information [file 41598_2017_527_MOESM1_ESM.pdf]

Supplementary Information

For submission: *Scientific Reports*

Metabolomics reveals biotic and abiotic elicitor effects on the soft coral *Sarcophyton ehrenbergi* terpenoid content

Mohamed A. Farag[1*], Dalia A. Al-Mahdy[1], Achim Meyer[2], Hildegard Westphal[2,3], Ludger A. Wessjohann[4]

[1]Pharmacognosy department, College of Pharmacy, Cairo University, Cairo, Egypt, Kasr El Aini st., P.B. 11562.

[2]Leibniz Center of Tropical Marine Ecology, Fahrenheit Str.6, D-28359 Bremen, Germany

[3]Bremen University, Germany

[4]Leibniz Institute of Plant Biochemistry, Dept. Bioorganic Chemistry, Weinberg 3, D-06120 Halle (Saale), Germany

*Corresponding author: Dr. Mohamed A. Farag
E-mail: Mohamed.farag@pharma.cu.edu.eg, mfarag73@yahoo.com, Tel: +011-202-2362245, Fax: +011-202-25320005

Supplementary Fig. S1 Quantum yields of photosystem II, Y, in corals exposed to different elicitors at 0.1 mM post 24 h , 0.1 mM post 48 h , 1 mM post 24 h & 1 mM post 48 h of elicitation as measured by pulse amplitude modulated (PAM) PAM measurement. For wounding and control un elicited corals, samples were harvested at 24 and 48 h . Results are average of 3 independent measurements.

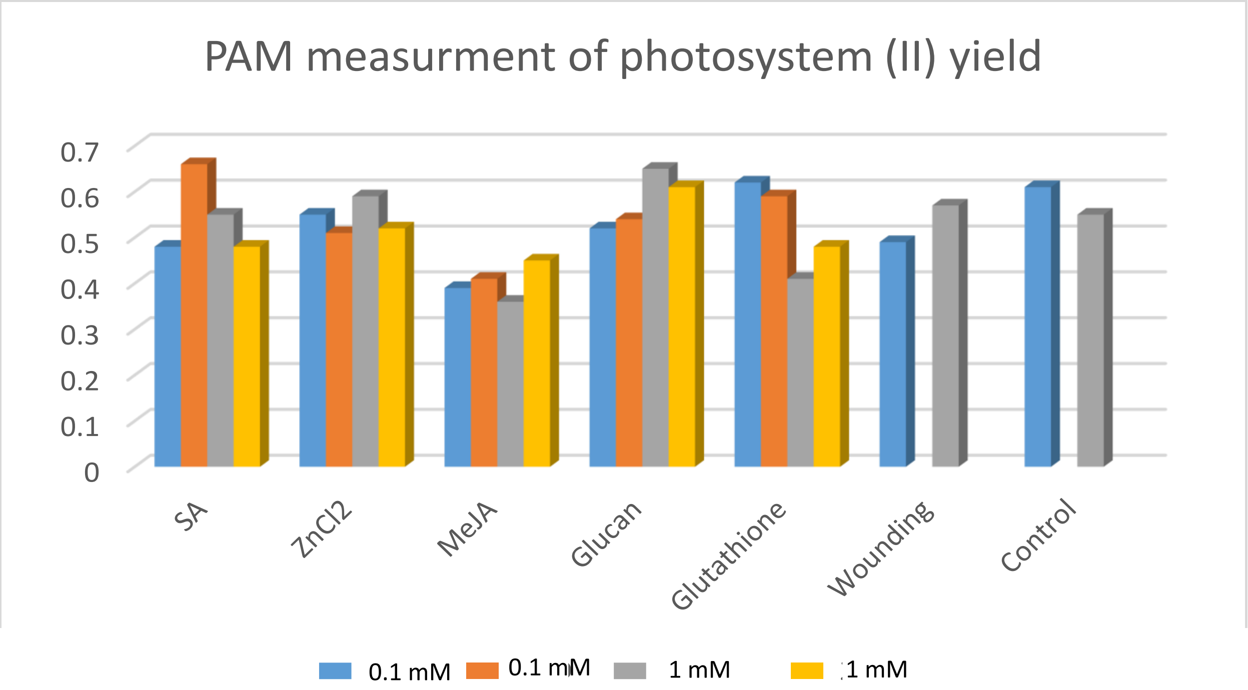

Supplementary Fig. S2 Heat map representation of hierarchical clustering (HCA) of UPLC-MS extracted metabolites from *S. ehrenbergi* elicited with methyl jasmonate (MeJA), ZnCl2, *β*-glucan, salicylic acid (SA), and glutathione at 0.1 and 1 mM and after wounding, harvested at 0 h (before), 24 h and 48 h post elicitation.

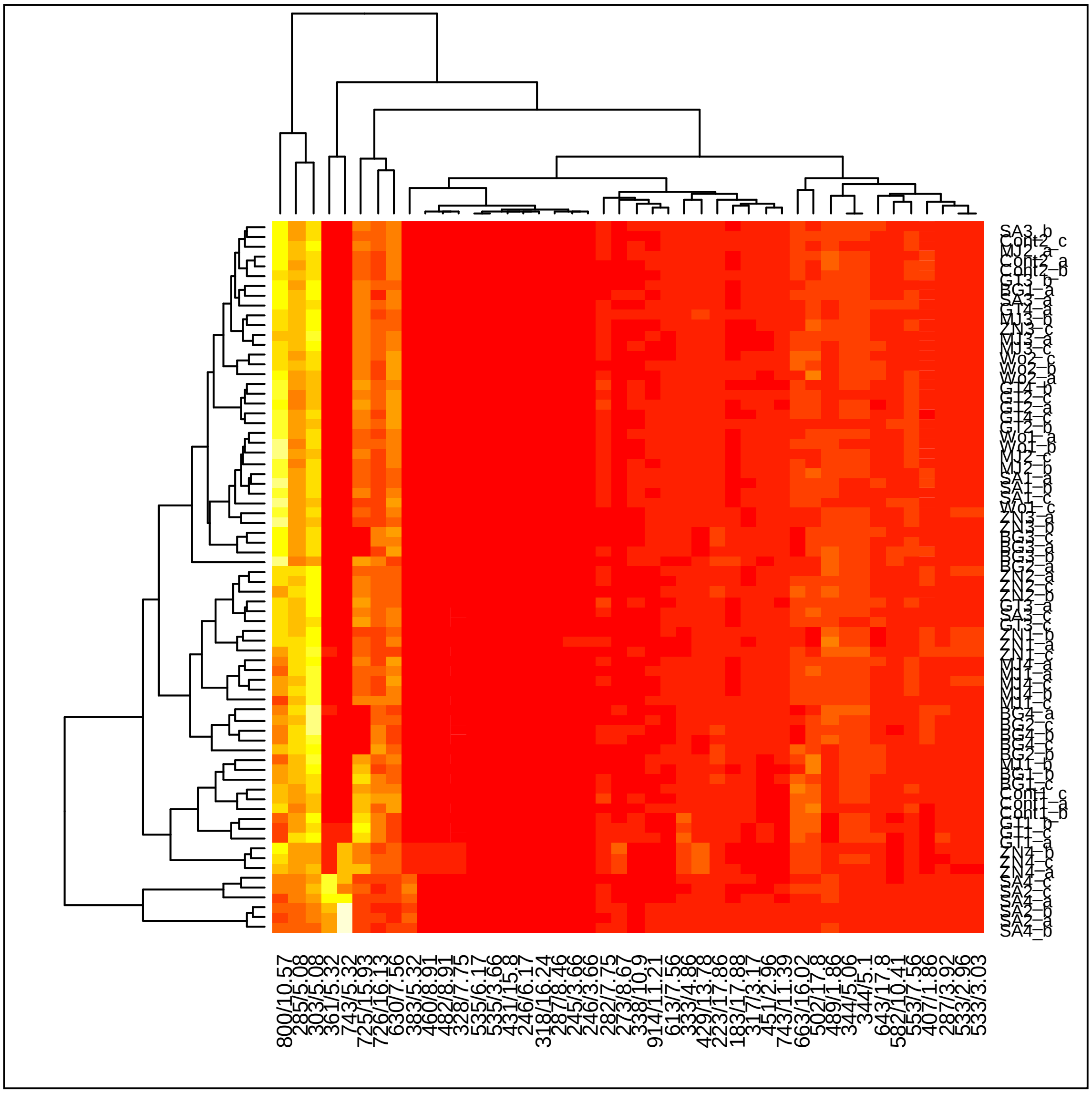

Supplementary Fig. S3 Reconstructed ion chromatogram for*m/z* 361 of sarcophytnolide in extractsof the soft coral *S. ehrenbergi*, elicited with salicylic acid (**A**), $ZnCl_2$ (**B**) at 48 h post elicitation and untreated control (**C**). Inset in panel A shows the structure and MS spectrum of sarcophytonolide I, the compound associated with *m/z* 361 base peak.

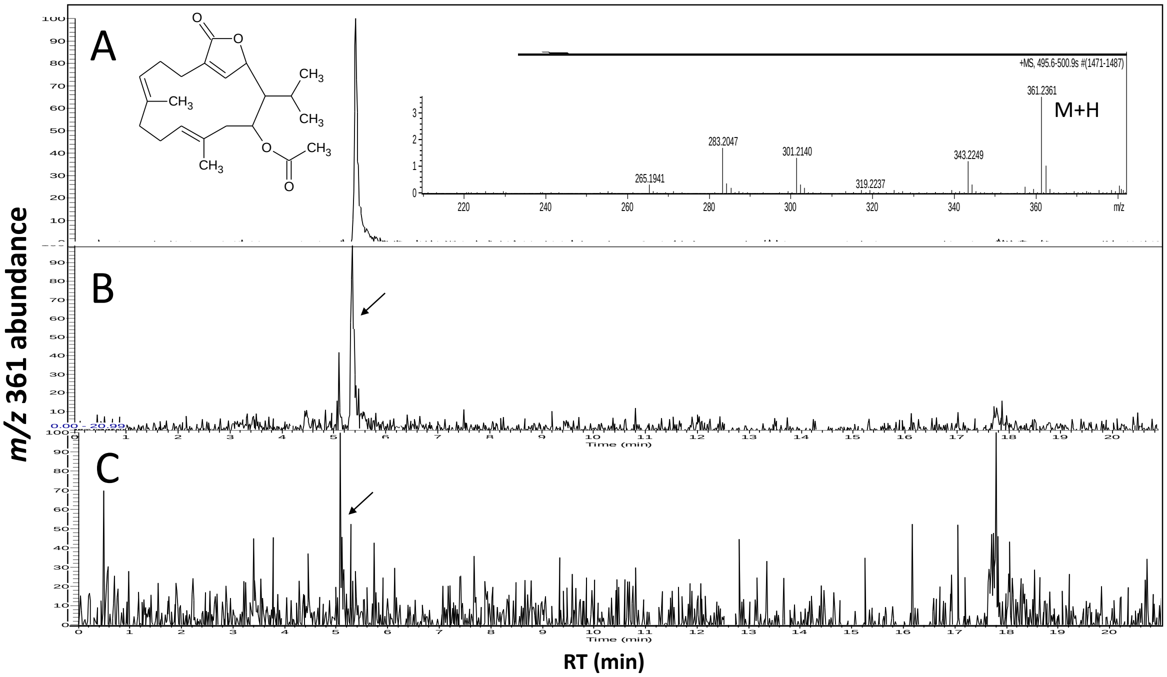

Supplementary Fig. S4 Pie chart showing metabolite classes percentile in zooxanthellea (**A**) versus corals tissue (**B**) as analyzed using GC-MS with silylation. Note the increment in sugar levels reaching 22% in zooxanthellae compared to coral tissue present at 3%.

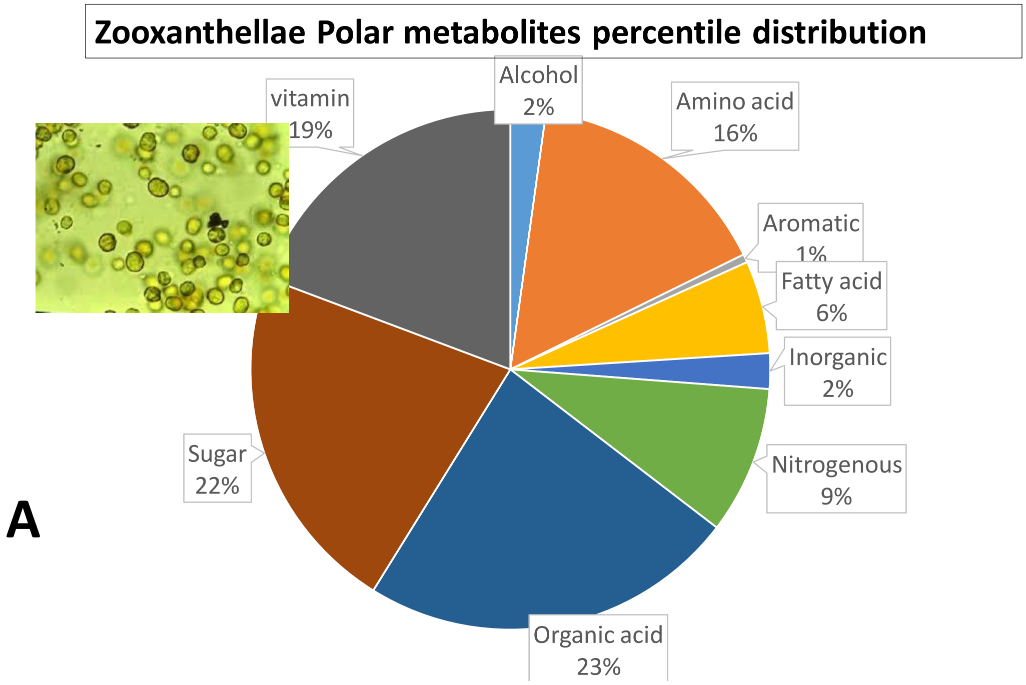

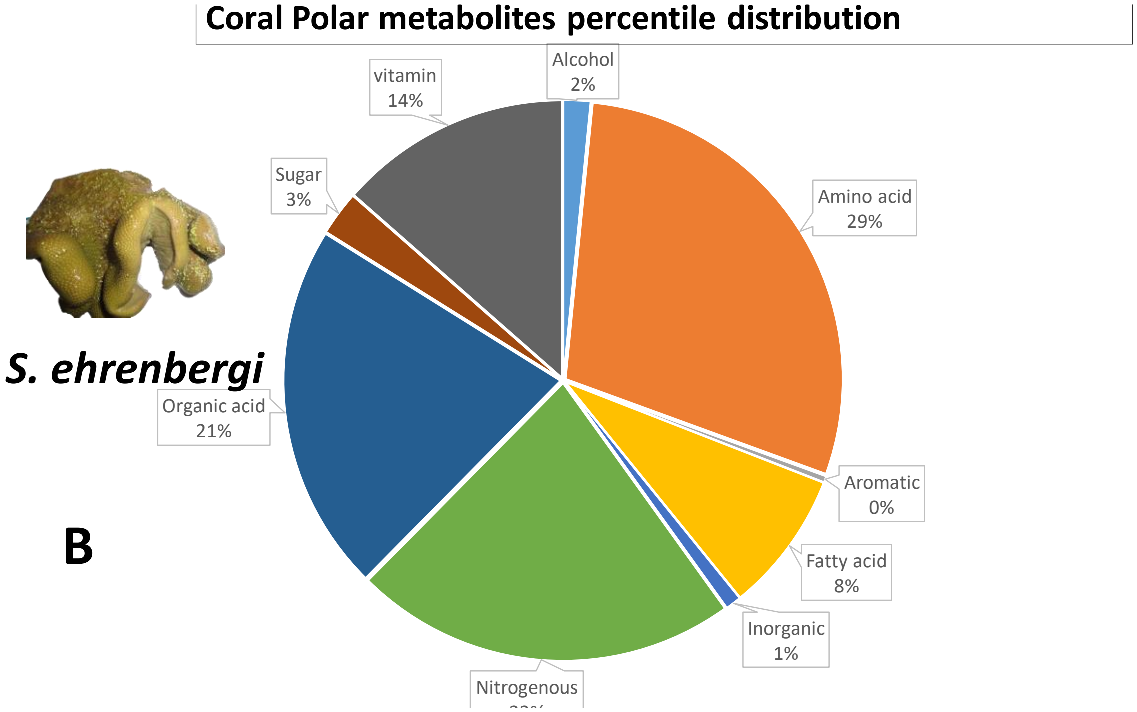
Coral Polar metabolites percentile distribution
S. ehrenbergi
B
Alcohol 2%
Amino acid 29%
Aromatic 0%
Fatty acid 8%
Inorganic 1%
Nitrogenous
Organic acid 21%
Sugar 3%
vitamin 14%

Supplementary text for UPLC-MS data extraction using XCMS

```
library(xcms)

setwd("C:\\Mohamed\\Sarcophyton_files")

lpath<-"."

files <- list.files(lpath, full.names = T, pattern = ".cdf", recursive = T)

length(files)

## Parameters for Peak Picking

# Signal/Noise Ratio

   snthr=3

  xset <- xcmsSet(files=files, method='centWave', ppm=25, peakwidth=c(5,12), snthr=snthr,

 verbose.columns=F, scanrange=c(500,2100), prefilter=c(3,500),nSlaves=10)

# scanrange = c(180, 2070) <<- 60.8 .. 697.4 sec ##

file='xset'              # Name of files

save(xset,file=file)

xset_group1 <- group(xset, minfrac = 1, bw = 5, mzwid = 1, max = 50)

xset_retcor1 <- retcor(xset_group1, plottype ="mdevden", span = 1, missing = 1, extra = 1)

xset_group2 <- group(xset_retcor1, minfrac = 1, bw=2, mzwid=0.05)

xset_filled <- fillPeaks(xset_group2)

save (xset_group1, xset_retcor1, xset_group2, xset_filled, file="full_sn3.Rdata")

load("full_sn3.Rdata")

values <- groupval(xset_filled, value="into")

dim(values)

write.csv(values, file="artichoke_sn3.csv")
```
